# Redox theory of aging: implications for health and disease

**DOI:** 10.1042/CS20160897

**Published:** 2017-06-30

**Authors:** Young-Mi Go, Dean P. Jones

**Affiliations:** Division of Pulmonary Medicine, Department of Medicine, Emory University, Atlanta, GA 30322, U.S.A

## Abstract

Genetics ultimately defines an individual, yet the phenotype of an adult is extensively determined by the sequence of lifelong exposures, termed the exposome. The redox theory of aging recognizes that animals evolved within an oxygen-rich environment, which created a critical redox interface between an organism and its environment. Advances in redox biology show that redox elements are present throughout metabolic and structural systems and operate as functional networks to support the genome in adaptation to environmental resources and challenges during lifespan. These principles emphasize that physical and functional phenotypes of an adult are determined by gene–environment interactions from early life onward. The principles highlight the critical nature of cumulative exposure memories in defining changes in resilience progressively during life. Both plasma glutathione and cysteine systems become oxidized with aging, and the recent finding that cystine to glutathione ratio in human plasma predicts death in coronary artery disease (CAD) patients suggests this could provide a way to measure resilience of redox networks in aging and disease. The emerging concepts of cumulative gene–environment interactions warrant focused efforts to elucidate central mechanisms by which exposure memory governs health and etiology, onset and progression of disease.

## Introduction

### Redox theory of aging

“Aging is a decline in plasticity of genome–exposome interaction that occurs as a consequence of differentiation and exposure memory systems.”

The redox theory of aging [1] was developed in response to improved understanding of oxidative stress [2] and advances in central redox theory outlined in the redox code [3,4]. The present article provides an update addressing the implications of redox theory in health and disease. We start with a summary of progress and refinement in the definition of oxidative stress after large-scale, double-blind free-radical scavenging antioxidant trials failed to show health benefits in humans. We briefly summarize the redox code, four principles by which oxidation–reduction (redox) mechanisms support life, and extend these concepts to the redox theory of aging. The redox theory was originally conceived as an extension of the redox hypothesis of oxidative stress, an alternative to free radical mechanisms of oxidative stress [5].

As the main points of the redox theory of aging, we discuss the importance of redox networks as an interface between an individual and his/her environment [6] and the evolution of exposure memory systems to allow animals to adapt to environmental conditions during lifespan to enhance survival and reproductive advantage [1]. The consequence of adaptation to lifelong exposures is a decline in flexibility and adaptability that underlies the theory.

A section is then provided on early life exposures as critical for physical and functional structures of adults, followed by a section on the importance of trace metals, some of which accumulate throughout life and cause progressive disruption of redox networks. This is followed by recent results suggesting that health of redox networks can be measured in terms of the ratio of the disulfide cystine (CySS) to the thiol glutathione (GSH), a ratio that predicts death as an outcome in coronary artery disease (CAD) patients [7]. Additional data are included to suggest that age-associated changes in redox network structures occur with many disease processes, including Type 2 diabetes, non-alcoholic fatty liver disease, atrial fibrillation, and other proinflammatory or profibrotic diseases. A following section briefly addresses the implications of lifelong accumulation of exposure memory in regenerative medicine, a rapidly developing area of innovative therapeutics.

Finally, we summarize and extend these concepts to complex systems approaches in medicine. This includes introduction of cumulative gene–environment interactions during lifespan as a central logic to complex systems research. This is part of a systematic effort to understand lifelong exposures in human exposome research [8,9] and will advance understanding of lifelong exposures in health and disease outcome. Additional study of plasma CySS and CySS/GSH as measures of redox network health could yield new ways to measure and manage resilience during aging. We conclude with the need for development of a hierarchical set of principles linking exposure memory to health outcome as a way to guide personalized health and disease prevention strategies.

## Oxidative stress

**Figure 1:**
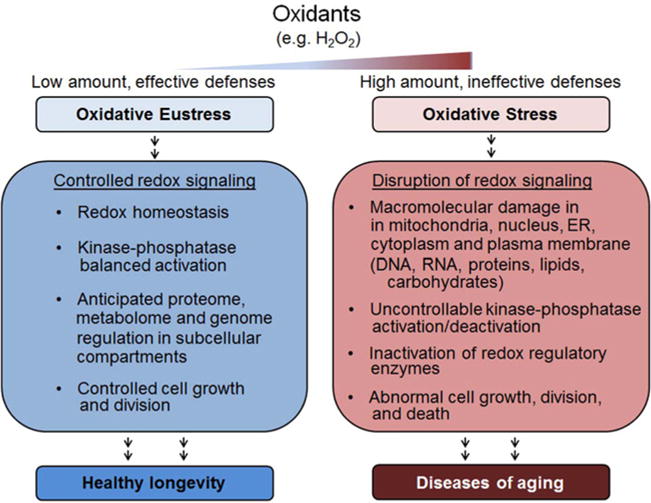
Oxidative stress and oxidative eustress within redox biology Advances in oxidative stress and redox signaling have led to improved definitions for considerations in health and disease. Oxidative stress is defined in a pathologic sense, while physiologic oxidant production is termed oxidative eustress. Redox biology embraces the continuum of oxidation–reduction reactions in normal biology and pathology, including non-enzymatic as well as enzymatic reactions. This broader view recognizes that an individual has continuous environmental interactions that have major impact on health and disease.

In 1985, Helmut Sies defined oxidative stress as an imbalance between prooxidants and antioxidants that resulted macromolecular damage [10]. The concept was popularized in news media and publications for non-scientific audiences and fueled development of a multibillion dollar antioxidant supplement industry. More than a billion dollars was invested in research to test antioxidants, and these generally showed that supplementation with free radical scavengers failed to provide health benefits. This caused confusion in the field and also contributed to a transition from oxidative stress of the early 1980s to the contemporary view of oxidative stress, as recently reviewed [11]. In the current definition, two aspects of oxidative stress are recognized, one with the original concept of macromolecular damage and the subsequently recognized disruption of redox signaling and control mechanisms leading to diseases of aging (Figure 1). The other encompasses physiologic levels of oxidant production, termed ‘eustress’, contributing to healthy longevity (Figure 1). The distinction between deleterious processes of oxidative stress and physiologic processes of eustress is important to maintain clarity in discussions of the broad range of oxidative reactions in redox biology. In the following sections, we discussed two aspects of oxidative stress further as the critical factors leading to differential aspects/consequences of aging and disease. Sies’s 1985 definition of oxidative stress included both 1-electron (free radical) and 2-electron (non-radical) oxidants; subsequent research often referred to reactive oxygen species (ROS) without discrimination of radical species, such as superoxide anion radical, from non-radical species, such as H_2_O_2_. The use of term ‘ROS’ is now discouraged, with the suggestion that specific oxidant names be used when possible and the general term ‘oxidant’ be used at other times [11]. Similarly, ‘antioxidants’ is a general term that includes different types of antioxidant chemicals, such as free radical scavenging vitamins (vitamin C and vitamin E) [12,13], singlet oxygen quenching dietary chemicals such as lycopene [14], and therapeutic thiol antioxidants such as *N*-acetylcysteine [15]. Science and medicine are best served by use of terminology defining specific antioxidants or types of antioxidants.

Two points are of central importance for the current discussion of the redox theory of aging and implications in health and disease. First, a substantial wealth of observational data shows evidence for oxidative stress for most major causes of human morbidity and mortality (Table 1). Thus, regardless of any specific arguments for or against oxidative stress [2], this data cannot be ignored. Second, all aspects of humans depend upon oxidation of foodstuffs for energy and maintenance of cellular NADPH pools to support detoxification and protection against environmental threats. Consequently, whether oxidative stress occurs is not a critical question; instead, critical questions are how to identify critical dysfunction of oxidant systems and how to develop useful interventions to minimize or reverse associated disease processes.

**Figure 2:**
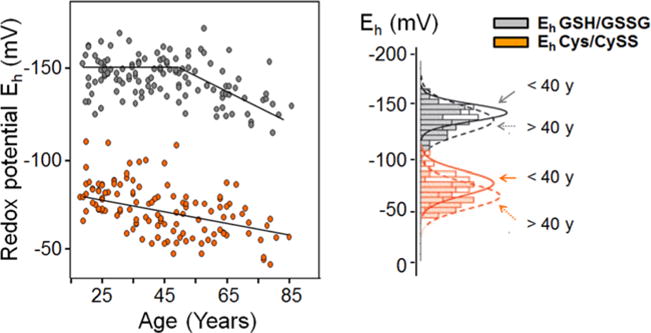
Plasma glutathione/glutathione disulfide (GSH/GSSG) and cysteine/cystine (Cys/CySS) redox potentials in humans Results show that these redox couples are not equilibrated and become oxidized with age [23,32,78]. The lack of equilibration of these systems implied that protein thiol/disulfide systems also exist in a non-equilibrium state, and this was subsequently confirmed (see text). Cross–sectional and longitudinal studies in humans showed oxidation of thiol/disulfide systems with age, suggesting that progressive changes occur due to lifelong interaction of individuals with environment. *E*_h_ is the steady-state redox potential calculated from the measured concentrations using the Nernst equation [78].

Denham Harmon proposed a free radical theory of aging in the 1950s [16], and observational studies and small interventional studies accumulated to justify large-scale double-blind interventional trials for many chronic and age-related diseases [17,18]. By the early 2000s, however, results from a sufficient number of these studies had accumulated to show that little to no significant health benefits occurred from supplementation with free radical scavenging antioxidants in humans [19,20]. Also by 2000, accumulating evidence supported a function for low concentrations of the oxidant H_2_O_2_ in redox signaling mechanisms [21,22]. Additionally, at about the same time, we found that the most abundant low molecular weight thiol/disulfide couples in plasma, glutathione/glutathione disulfide (GSH/GSSG), and cysteine/cystine (Cys/CySS) were not in thermodynamic equilibrium (Figure 2) [23]. Together, these results spawned considerable research into new directions of oxidative stress research.

Our finding of disequilibrium of GSH/GSSG and Cys/CySS led us to speculate that Cys residues in proteins could also be kinetically limited and this could be used for redox control [24]. Specifically, the reactivities of most thiols in proteins are similar to the thiols of GSH and Cys, so protein thiols are also likely to be kinetically controlled. We subsequently found this to be true in targeted studies of the thioredoxin (Trx)-1 system [5,25] as well as in the mitochondrial Trx-2 system [26,27]. More recent mass spectrometry studies also showed this to be correct for the steady-state oxidation of hundreds of specific protein cysteine residues in cell culture [28,29] and in mouse tissues [30]. Thus, in the period between 2000 and today, a major shift has occurred in the focus of oxidants/antioxidants balance from radical/radical scavenger balance to thiol/disulfide balance.

Together with recognition that plasma GSH/GSSG is oxidized in association with age and with Type 2 diabetes [31], knowledge of the disequilibrium of the GSH and Cys systems in plasma led to experimental studies of effects of variation in extracellular thiol/disulfide systems in human cells. Remarkably, cells in culture adjusted extracellular Cys/CySS redox potential (*E*_h_, calculated from concentrations with the Nernst equation) [32] to the value found in young healthy human plasma [32]. Exposure to more reducing conditions caused cells to proliferate more rapidly while exposure to more oxidizing conditions caused cells to proliferate more slowly [33,34] and have increased sensitivity to apoptosis [34]. Thus, the studies established a fundamental importance of thiol/disulfide redox control in the functions of human cells. Moreover, the results showed that thiol/disulfide systems activate redox mechanisms previously attributed to oxidative stress. In 2002, we found that human plasma GSH and Cys redox couples were oxidized at different rates as a function of age [32]. The lack of balance between the GSH and Cys thiol antioxidant systems, the failure of the free radical scavenger trials, and the accumulating knowledge of thiol systems in redox signaling led to the proposal that oxidative stress should be redefined in terms of disruption of redox signaling and control [35]. The most critical aspect of this transition was the recognition of kinetic limitations in thiol/disulfide systems. Under all aerobic conditions, thiols undergo oxidation that is balanced in the steady state by reduction systems.

## Transition to redox biology

A focus on oxidative stress transitioned to a more general focus on redox biology as knowledge of redox signaling mechanisms improved [35–38] and redox proteomics methods began to reveal the organization structure of the redox proteome [29]. An important contribution to this transition occurred as functions of NADPH oxidases in different organ systems and diseases was elucidated. In this, the term ‘oxidative stress’ was sometimes misused in that the oxidants produced were physiologic and not pathologic. Recently, the term ‘oxidative eustress’ has been recommended for use to describe beneficial production of oxidants so that the term ‘oxidative stress’ retains its original reference to adverse processes (Figure 1).

**Figure 3:**
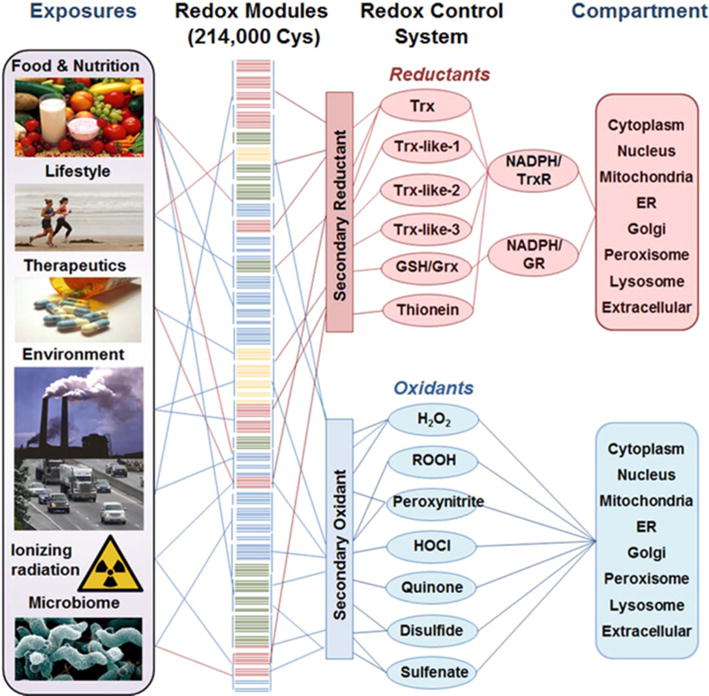
Protein thiols exist within modules having similar redox behavior The human genome encodes 214,000 Cys in proteins that vary in percent oxidation according to functional pathways (Redox Modules, center) [28]. The steady-state oxidation/reduction of these Cys is controlled by opposing oxidative (bottom right) and reductive (top right) systems. A bilateral scale-free network structure involves subcellular compartments, and primary reductant and oxidant systems only require one additional layer of secondary reductants and oxidants to provide selective control of each protein Cys [36]. Evidence for specific systems supports this redox network structure. These redox network structures are stable and protect the individual against a broad range of oxidative exposure from diet and environment (left). Image credits: Smokestacks from Alfred Palmer—US Library of Congress CALL NUMBER LC-USW36-376, reproduction number LC-DIG-fsac-1a35072; food photo by Peggy Greb, USDA Agricultural Research Service; Prozac photo, Tom Varco (tomvarco@gmail.com); runners: Mike Baird from Morro Bay, USA (http://www.flickr.com/photos/mikebaird/3539161615/) [CC BY 2.0 (http://creativecommons.org/licenses/by/2.0)], via Wikimedia Commons; Campylobacter jejuni photo by De Wood and digital colorization by Chris Pooley.

Along with improved understanding of oxidant production in redox signaling, studies of Trx systems in cell nuclei, cytoplasm, and mitochondria showed that subcellular compartments are maintained at different thiol/disulfide steady states [39]. The mitochondrial and cytoplasmic steady states also differ for GSH/GSSG, and the cytoplasmic Cys/CySS differs from the steady states for GSH/GSSG and the Trx system [39,40]. Thus, the results emphasize that kinetic limitations are widespread in thiol/disulfide systems and that differences exist in the characteristics of the central redox hubs [36]. A possible organizational structure is illustrated in Figure 3, based upon the scale-free hierarchical network proposed for metabolomics [41]. In this global view, environmental exposures selectively affect subnetworks of redox-sensitive elements, conceptualized as ‘redox modules’. These redox modules are maintained in steady state by endogenous reduction and oxidation systems. Each subcellular compartment has sources of reductants and oxidants. These include a relatively small number of NADPH-dependent reductases and a relatively small number of oxidants. In a bilateral hierarchical structure, these require only one additional level of secondary reductants and secondary oxidants to provide selective regulation of each of the 214,000-specific Cys encoded in the human genome [36]. Studies of proteins with nuclear import machinery shows selectivity in redox interactions [42], supporting this modular network structure. Similarly, import of proteins into mitochondria shows selectivity in redox reactions [37], and protein processing within the endoplasmic reticulum shows selectivity [43]. Targeted studies further show specificity in redox systems during signaling. For instance, redox signaling by NADPH oxidase was found to involve H_2_O_2_ and occur without detectable changes in either the Trx or GSH/GSSG systems [44]. KGF signaling in keratinocytes occurred with selective oxidation of cytoplasmic Trx1 without oxidation of mitochondrial Trx2 [45]. In contrast, TNF-α triggered oxidation of mitochondrial Trx2 without oxidation of cytoplasmic Trx1 [46]. Additionally, selective generation of H_2_O_2_ in cell nuclei by nuclear-targeted D-amino acid oxidase resulted in localized nuclear thiol oxidation without cytoplasmic oxidation [47]. Application of mass spectrometry-based redox proteomics has extended these concepts to show that the redox network structure has a central function in the tolerance and adaptability of an organism to diet and environmental challenges (Figure 3) [29]. Recognition of this redox interface between an individual and its environment [48] provided important background to formulation of the redox principles of the redox code.

## The redox code

**Figure 4:**
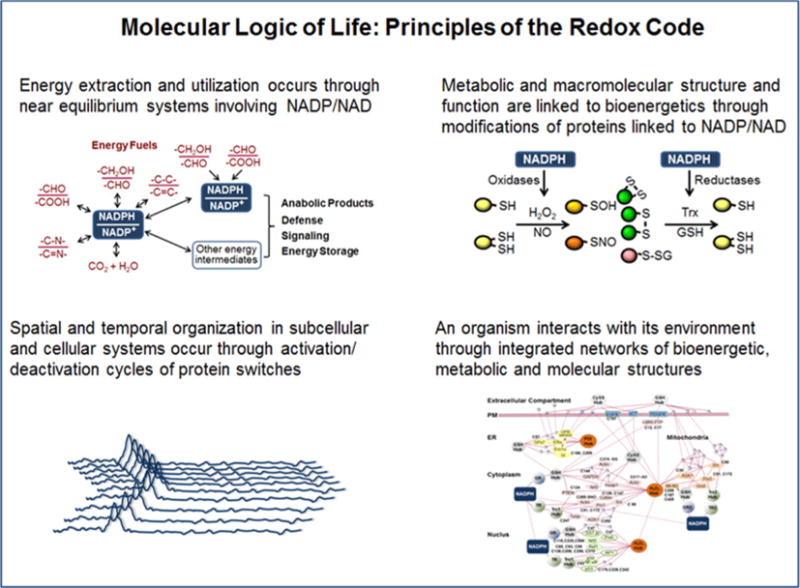
Four principles of the redox code The molecular logic of life includes four redox principles for organization of bioenergetics, metabolism, and macromolecular structure and function. In this structure, energy is derived from oxidation reactions involving NAD and NADP systems. The energetic systems maintain metabolic and macromolecular organization through molecular switches in the proteome, and activation/deactivation of these switches provides spatial and temporal organization in complex multicellular systems. The overall network structure provides an adaptive interface for an organism to maintain delineation and interact with its environment. From [3].

The redox code (Figure 4) is a set of principles for redox organization and function of metazoans [3]. Living organisms exist in stable thermodynamic disequilibrium with four basic characteristics: metabolic and structural organization, delineation from environment through semipermeable barriers, reproduction, and extraction and use of energy to maintain the other three characteristics. The first principle of the redox code is that energy systems are maintained at near-thermodynamic equilibrium through high-flux oxidation–reduction (redox) reactions involving NAD and NADP systems. The second principle is that these high-flux systems are connected to macromolecular structure and function through an array of reversible, kinetically-controlled switches in proteins involving oxidation, acetylation, phosphorylation, methylation, and other modifications. The third principle is that reversible activation/deactivation of these switches support spatial and temporal signaling and organization to control structure and function in cell differentiation and development. The fourth principle is that these interactive systems function as networks at molecular, cellular, and organ system levels to allow an individual genome to adapt during lifespan to environmental resources and challenges [3].

## The redox theory of aging

**Figure 5:**
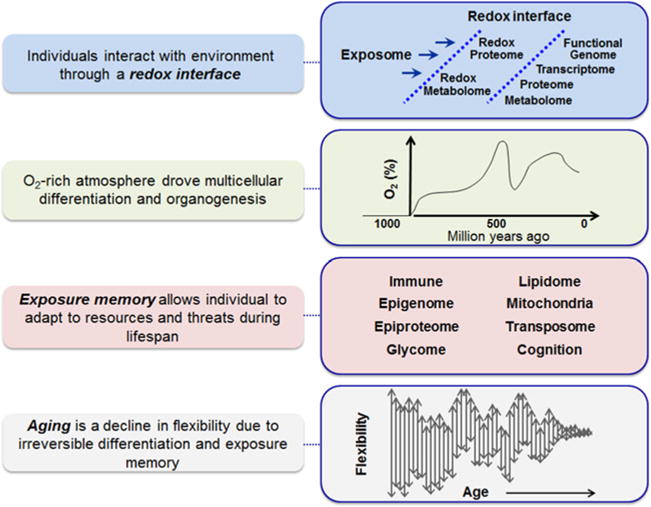
The redox theory of aging The principles of the redox code (Figure 4) provide a basis for a new interpretation of aging. The redox metabolome and redox proteome provide an important interface between an individual and his/her environment [6]. An increase in atmospheric O_2_ enhanced the magnitude of the intracellular/extracellular redox gradients and provided driving forces for multicellular differentiation and evolution of complexity in metazoan speciation. Genetic systems evolved programs to support this speciation, with the important characteristics that the systems provided memory systems to facilitate adaptation to environment during the lifespan of an individual. Within the overall redox network structure, accumulation of adaptive responses during development and lifelong exposures results in decreased adaptability over time. Aging is the decline in adaptability due to irreversible characteristics acquired in response to exposures during differentiation, maturation, and subsequent adult life. From [1].

The redox theory of aging was developed from the redox hypothesis of oxidative stress [5], a hypothesis to explain oxidative stress without requirement for free radicals. After more than 50 years of study, lack of firm support for the free radical theory of aging [16,49–51] provided impetus to extend the radical-free concepts of oxidative stress to formulate a radical-free theory of aging [1]. The theory (Figure 5) acknowledges that networks of the redox proteome and metabolome serve as an adaptive interface [29] to allow an individual to adapt during lifespan to environmental resources and challenges. The redox modifications of the proteome provide a system to sense, avoid, and defend against oxidants and other toxic chemicals from the environment. The theory considers the rise in atmospheric O_2_ beginning 2 billion years ago as a driving force for improved energy extraction machinery and evolution of multicellularity to avoid O_2_ toxicity. The theory accommodates the increase in Cys content of the proteome with evolution of complexity [52] to improve tolerance to different oxidative environments. The theory also accounts for genetic systems directing cellular differentiation and organ development as mechanisms to improve adaptability to the O_2_-rich atmosphere.

The theory thus interprets genetic systems for development and response to environment as exposure memory systems to allow an individual to adapt during lifespan to environmental resources and challenges [1]. Genetically encoded memory systems are emphasized because they were essential for transition of unicellular organisms into differentiated multicellular organisms. Other forms of exposure memory occur, such as changes in membrane lipid composition due to dietary lipid intake and variations in metal-bound structures and reactivities dependent upon metal ion exposures. Systematic studies are needed to evaluate contributions of different memory systems to long-term adaptability.

A natural consequence of use of memory systems for adaptability is that response to one challenge can decrease adaptability to other challenges. Over time, the use of these differentiation and environmental response systems results in decreased flexibility to accommodate additional environmental challenges. Therefore, aging is a decline in plasticity of gene–environment interactions that occurs as a consequence of differentiation and exposure memory (Figure 5). The integrated redox networks that are essential for cellular energetics, metabolic and structural organization, defense against environmental challenges, and reproduction, ultimately fail because of environmental challenges that cannot be accommodated [1]. Epigenetics and immune systems provide examples of systems that are used to provide memory of prior exposures. These systems allow a genome to adapt to environmental exposures during lifespan. Irreversible changes due to the operation of these systems ultimately limit their beneficial functions. Similarly, telomere shortening, cellular senescence, and stem cell exhaustion reflect cumulative memory of prior differentiation and responses to exposures.

The redox theory accounts for other characteristics of aging. For instance, ongoing oxidative challenges are opposed by responses of the thiol reducing systems. As any component of the redox network system becomes compromised, the entire network responds and becomes less tolerant to additional challenge. Thus, increased biomarkers of oxidative stress, such as hydroxynonenal and reactive carbonyls, and other general biomarkers of oxidative stress, are increased [53,54] even though they may not be directly related to the factors compromising the redox network structure. Oxidative modifications of slowly turning over proteins, as well as membrane lipids and DNA, create a burden decreasing the flexibility of the network to respond to additional challenges. Similarly, oxidative reactions as well as protein modifications from reactive carbonyls contribute to other hallmarks of aging, such as accumulation of macromolecular aggregates and failure of proteostasis, intercellular communication, and protective barriers. With this theory, genomic instability can occur due to failure of the redox network structures, including active defenses, repair systems, and adaptive memory systems.

Redox theory also incorporates mitochondrial support of bioenergetic functions in all O_2_-requiring cells, including maintenance of central ATP and NADPH pools. While ATP requirements are well known, less attention is given to NADPH, the primary reductant to maintain redox networks and also a primary precursor for H_2_O_2_ generation to maintain redox networks [3]. NADPH supply rates vary among cell types but are often much slower than rates of NADH supply to support mitochondrial ATP production. In liver, for instance, the maximal rate of NADPH supply is only 20% of the rate of NADH supply [55]. Furthermore, when mitochondrial ATP supply is insufficient to meet demand, glycolysis is stimulated. Both glycolysis and NADPH supply depend upon glucose-6-phosphate, and during hypoxia, glucose-6-phosphate is preferentially used for glycolytic ATP production at the expense of NADPH supply by the pentose phosphate pathway [55]. Detailed information is not available about the relative sensitivities of other systems controlling reversible switches within the proteomic networks. In particular, acetylation/deacetylation mechanisms controlling sirtuins depend upon the NADH/NAD system. Additionally, acetylation requires acetyl-CoA, and methylation requires *S*-adenosylmethionine; both of these precursors are linked to cellular energetics. Consequently, conditions that limit mitochondrial ATP production have a widespread impact on NADPH supply and other systems essential for maintenance of the redox network structures [55].

This dependence of both NADPH supply and ATP supply upon common precursors links bioenergetic responses to diet and oxidants to the flexibility of redox network structures. In other words, impaired mitochondrial ATP supply stimulates glycolysis, thereby limiting the pentose phosphate pathway supply of NADPH needed to maintain GSH and Trx functions. This links energy supply and antioxidant systems in their functions to accommodate environmental challenges. Growth factor signaling and nutrient regulation are ultimately linked to the same network structures controlled by mitochondria. The integration of these systems with epigenetic regulation, DNA repair, immunity, antioxidant defenses, and maintenance of cell populations leads to the perspective that strategies to delay aging, prevent and manage disease must address the cumulative memory of exposures as they affect mitochondrial function and redox control. At the global level, a primary focus for disease prevention must include the cumulative impact occurring at this genome–exposome interface [6]. For management of disease, a primary focus must include targeted support for these hubs controlling the steady-state dynamics of the redox networks [36]. For rejuvenation following loss of functions, a primary focus must be reversal of exposure memory that caused the loss of resilience [56].

## The redox interface in disease risk

### Lifelong consequences of early exposures

The implications of redox theory for disease prevention are closely aligned with developing concepts of the human exposome [8]. Most human disease is attributed to cumulative lifelong exposures [57]. The foremost implication is that early life exposures affect lifelong health because the signaling mechanisms in cellular differentiation and organogenesis were driven by redox mechanisms associated with the dramatic rise in atmospheric O_2_ early in metazoan evolution. In a practical sense, there are needs for greater precision in understanding the key exposures and windows of vulnerability, not just for severe, early onset disease but also for risk of chronic disease. Systematic studies are not available for a broad range of exposures, but epidemiologic and model system studies show early life exposures affect adult disease [58,59]. Efforts to measure human exposures and associated health outcomes will be greatly facilitated if the international research and technology communities embrace a ‘Human Exposome Project’ [8,60–63] (http://humanexposomeproject.com/) to complement the Human Genome Project (https://en.wikipedia.org/wiki/Human_Genome_Project) in advancing underlying causes of disease.

Several environmental agents, especially endocrine disruptors [64] and obesogens [65] have received considerable attention. An extensive list of poorly metabolized, persistent chemicals, including plasticizers, flame retardants, and insecticides, act as agonists and antagonists in receptor signaling. In the context of development, disrupted signaling can have lifelong consequences. Details are beyond the scope of the present article, but the implications are extensive. An example from the literature on smoking in pregnancy serves to illustrate the point. Nicotine binds to nicotinic acetylcholine receptors, including α7 nicotinic acetylcholine receptors directing lung organogenesis [66]. In mouse studies, nicotine binding to the receptor during a critical developmental window increased airway length and decreased airway diameter, resulting in a persistent change in airway geometry and impaired lung function in the adults. This example emphasizes that the spectrum of impact of early exposures includes size and form of organ systems, as well as more commonly considered endocrine and immunologic responses. Many research programs are in place, such as HELIX (Human Early-Life Exposome), a European Union-funded project to integrate early life exposures and child health across Europe (http://www.projecthelix.eu), but the key point for contemporary medicine is that currently, there is no atlas linking early exposures to lifelong health and disease. Thus, an important implication of the redox theory is that there are needs to establish programs for ‘deep-sequencing’ of the human exposome [63], with the ultimate goal to be able to evaluate early exposures as beneficial or harmful in long-term health outcomes. Barriers include cost and lengthy longitudinal follow-up. Computational methods, such as a framework to address the large number (a million or more) human exposures [67], are beginning to provide ways to overcome the barriers, but will need to be extended to study of large populations and model systems.

### Lifelong accumulation of metals

A second important aspect of the redox interface involves environmental metals. Metal content in the soil varies considerably by geography, and metals entering the food chain reflect this variation. Several metals such as iron (Fe), copper, and manganese (Mn) are redox active and essential for bioenergetics and other metabolic functions. Others, such as zinc, are not redox active but essential in macromolecular structures. Others, such as cadmium (Cd), mercury, and lead, are toxic. For some metals such as Cd and Fe (in men and post-menopausal women), there are no effective elimination mechanisms.

The redox network structure is effective in accommodating moderate deficiencies of essential nutrients through widespread adjustments. For instance, decreased growth rate and decreased size can accommodate moderate deficiencies. Operation of a system at lower rates means that longer recovery time may be needed to deliver the same product, so response and recovery to stress can be delayed. But the key implication in redox theory is that excesses, which cannot be eliminated, are more disruptive by decreasing flexibility to adapt to other challenges. This is true for essential nutrients such as Fe and Mn, and also true for toxic metals such as Cd. A balloon provides a simple analogy. If inflated but not completely filled with air, the balloon is resilient to repeated deformation. If overfilled, however, the balloon has no remaining flexibility and easily fails. Redox systems with metals have this same character. With extensive number of relatively weak metal-binding sites in proteins, redox systems can accommodate a wide variety of metals and retain function. In redox theory, many of these binding sites are functional in the coupling of bioenergetics to macromolecular structure and function (second principle of the redox code) and show progressive impairment with excess. The major implication in health and disease is that excesses of metals must be avoided. This is true for individual metals including essential metals, and perhaps more importantly, is true for metals collectively.

## Plasma CySS/GSH as a mechanistic biomarker of death in coronary artery disease

The recent finding that elevated cystine/glutathione (CySS/GSH) ratio in plasma predicts death as outcome in CAD patients [7] provides some of the strongest evidence for the importance of redox networks in human health. This study followed a cohort of cardiovascular disease patients over 7 years and showed that the ratio of plasma CySS to GSH predicted all-cause mortality after adjustment for all other known risk factors. Consideration of redox control mechanisms allows interpretation of this finding in terms of integrated redox networks. Molecular O_2_ is the ultimate oxidant for maintenance of bioenergetic functions and also for maintaining H_2_O_2_ pools for the redox proteome networks. NADPH oxidase (Nox)-4 in mouse mitochondria is a source of H_2_O_2_ generation in cardiomyocytes and promotes aging [68,69], providing a possible mechanism for oxidation with age. H_2_O_2_ serves in intracellular communication and control of macromolecular structure and function but is present at nanomolar concentrations and cannot be measured in a practical way in patients. CySS also oxidizes protein thiols, however, and has an advantage that it is present at micromolar concentrations in tissues and plasma and is readily measureable. Thus, CySS provides a surrogate to evaluate overall oxidation *in vivo*.

NADPH is the ultimate reductant to maintain the steady state of the redox proteome, but like H_2_O_2_, is difficult to measure in patients. In tissues, NADPH supports Trx and GSH systems, which directly interact with the redox proteome to maintain redox networks. Trx is released into plasma under some conditions but does not preserve its redox function outside the cell. In contrast, GSH is transported into plasma to maintain an interorgan system for redox homeostasis. The plasma GSH concentration can therefore provide a surrogate for the NADPH systems in tissue, which maintain the stable, non-equilibrium steady states of the redox networks. GSH is released from cells as a function of cell concentration [70] and is only a minor component in human plasma. While tissue concentrations of GSH are in the millimolar range, human plasma contains only low micromolar GSH [32,71]. The low concentration in plasma has been an important limitation to its usefulness in clinical medicine but this limitation can be overcome with appropriate sample collection and processing procedures [72]. There is evidence that ratio of GSH/GSSG is important in platelet activation [73], but the GSH concentration in human plasma is typically 3- to 10-fold lower than plasma Cys [23] and 50- to 100-fold lower than albumin thiol concentration [74]. Thus, we interpret the plasma GSH mostly as a reflection of the health of NADPH-dependent reduction systems in tissues rather than supporting important functions in the plasma compartment.

**Figure 6:**
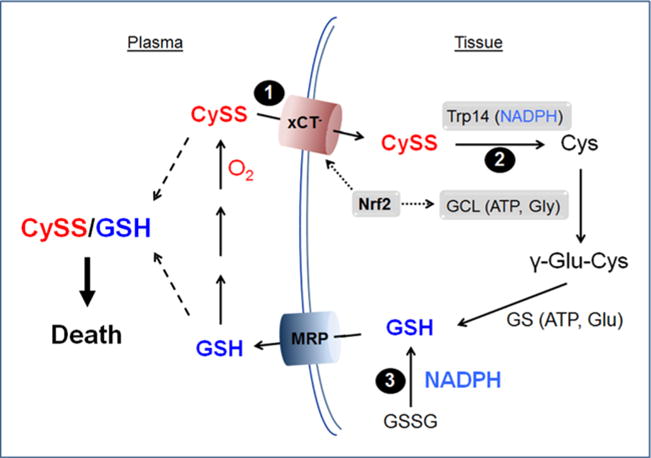
CySS/GSH as a mechanistic biomarker of the health of redox networks affecting age-related diseases This scheme describes possible control mechanisms for CySS/GSH that contribute to redox health, generalized from the finding that CySS/GSH is an independent predictor of CAD death outcome after control for all other known risk factors. (**1**) CySS is cleared from plasma by xCT^−^ and other transport systems. No direct pharmacokinetic analyses are available for CySS clearance, but indirect estimates show an apparent volume of distribution equal to the total body water. This implies that plasma CySS is a surrogate for whole body thiol/disulfide oxidation status. (**2**) Within tissues, Trx and GSH-dependent systems have low levels of CySS reductase activity [94], and recently, a Trx-related protein, Trp14, has been identified as a CySS reductase [95]. The kinetic characteristics of this system suggest that it may become saturated and have limited capacity at high physiologic concentrations of CySS. GSH is the major low molecular weight cellular thiol antioxidant that is synthesized from the amino acid cysteine in two ATP-requiring steps. The first step catalyzed by glutamate–cysteine ligase forms γ-Glu-Cys and the second catalyzed by GSH synthetase produces GSH. The first step is rate limiting with feedback inhibition of enzyme activity by GSH and transcriptional regulation by the Nrf2 system [92]. (**3**) GSH release from tissues occurs through ubiquitous Mrp (multidrug resistance-associated proteins) family transporters [187]. GSH release is concentration dependent and affected by multiple factors [187]. The present interpretation is that GSH in plasma provides a surrogate for tissue NADPH supply. NADPH is used to reduce GSSG to GSH and thereby maintain plasma GSH concentrations. GSH in human plasma is metabolized by two interconnected pathways, one involving hydrolysis by γ-glutamyltransferase (GGT) and dipeptidases to form Cys, which is oxidized to CySS, and the other involving thiol–disulfide exchange with CySS to form glutathione–cysteine disulfide, which is then hydrolyzed to form CySS [23,188]. CySS/GSH in plasma is correlated to CAD death outcome [7]. Not shown: Cys export from cells occurs at rates greater than GSH export, but plasma Cys/CySS redox potential is insufficient to reduce GSSG to GSH; Cys is generated from Met at rates greater than GSH synthesis; rates of Cys incorporation into protein exceed rates of GSH synthesis; rates of GSSG reduction to GSH exceed rates of GSH synthesis [189].

The mechanisms to control plasma CySS/GSH are summarized in Figure 6; long-term failure of these systems could contribute to the increase in CySS/GSH linked to death in the CAD patients. The central reactions controlling plasma CySS/GSH involve GSH export from tissue, with major contributions from liver and skeletal muscle, and CySS clearance by transporters, with xCT^−^ having a major contribution ((1) in Figure 6) [75,76]. The interorgan CySS → Cys → GSH → GSSG → CySS cycle is kinetically limited at multiple sites, with different mechanisms for thiol oxidation, reduction of CySS to Cys, and control of GSH levels [77].

CySS is the most abundant low molecular weight disulfide in human plasma, formed from the oxidation of amino acid, Cys or from the degradation of GSH oxidation products [23]. CySS is increased in human plasma in association with demographic factors and health behaviors, e.g. age [32,71,78,79], obesity [80], cigarette smoking [78,81], and alcohol abuse [32,82], and also with multiple disease processes, e.g. HIV-1 infection [83], carotid intima media thickness [84], endothelial cell function [85], Type 2 diabetes [31], and age-related macular degeneration [86]. Mechanistic studies have addressed the impact of elevated extracellular CySS on cellular functions. Most studies address steady-state CySS/Cys redox potential; in these studies, however, CySS is the most abundant variable. High CySS activates NF-κB signaling in mouse aortic endothelial cells, increases expression of cell adhesion molecules, and activates monocyte adhesion [78]. The process involves oxidation of integrins and other plasma membrane proteins [78], stimulation of mitochondrial oxidant production, and increase in expression of proinflammatory cytokines [87]. High CySS increased IL-1β in U937 monocytes [88] and IL-1β-related transcripts in THP1 monocytes [89]. High CySS inhibited proliferation in Caco2 cells [33] and retinal pigment epithelial cells [34] but stimulated proliferation in lung fibroblasts [90]. High CySS blocked TGF-α signaling in CaCo2 cells [91] and activated apoptosis in retinal pigment epithelial cells [34]. These studies show that elevated concentrations of CySS as found in CAD patients activate processes that contribute to many disease processes and adverse health outcomes.

### CySS/GSH as a mechanistic biomarker

Combining the results of the mechanistic studies with the results on death in CAD patients leads to the interpretation that CySS/GSH could be a mechanistic biomarker relevant to CAD outcome and also a more general measure of the health of redox networks affecting many age-related diseases. In this interpretation, CySS provides a measure of the oxidant burden in the redox network and GSH provides a measure of the NADPH-dependent reductive capacity. In Figure 6, critical steps are identified that are possible targets for intervention. Nrf2 controls expression of the CySS transporter xCT^−^, a transcription factor controlling many redox systems [92]. Nrf2 activity is controlled by interaction with actin-associated inhibitory binding protein, KEAP-1, and small Maf proteins in nuclei. Perhaps most importantly, the maximal inducible Nrf2 activity decreases with age [93]. Thus, development of approaches to control this activity may enable control of redox networks to protect against disease and disease outcomes.

Much less is known about the reduction of CySS after transport into cells. Kinetic studies for plasma CySS following consumption of a high sulfur amino acid meal showed that the volume of distribution is equivalent to the total body water, indicating that the rate of reduction of CySS is slow relative to the rate of uptake. Within tissues, Trx and GSH-dependent systems have low CySS reductase activity [94], and recently, a Trx-related protein, Trp14, has been identified as a CySS reductase ((2) in Figure 6) [95]. The dependence upon CySS concentration indicates that the reductase activity may become saturated at higher CySS concentrations and thus limit the capability to remove excess CySS. Thus, mechanisms to enhance expression or activity of this system could provide another potential target to maintain or improve redox networks.

Many approaches have been used to enhance GSH concentrations in model systems and in humans; results have been mixed and details cannot be provided here. Most importantly, the interpretation that circulating GSH is an indirect surrogate for tissue NADPH ((3) in Figure 6) implies that focus on GSH, per se, may not be the best strategy. A relatively small number of NADPH supply systems provide most NADPH in tissues. These include glucose-6-phosphate dehydrogenase (G6PD) and 6-phosphogluconate dehydrogenase (6PGD) in the pentose phosphate pathway, the NADP-dependent malic enzyme (ME1), NADP-isocitrate dehydrogenase (IDH2), and mitochondrial proton-translocating NAD(P)^+^ transhydrogenase (NNT). If limitation of the redox networks lies with the function of NADPH supply, then these would appear to be the most appropriate targets. The reductive hubs supported by NADPH may also be appropriate targets. Inhibition of the selenoproteins, Trx reductase 1 and 2, showed widespread protein oxidation [96]. These enzymes are sensitive to environmental toxicants such as Cd and reactive aldehydes like acrolein [97]. Interventions to enhance Trx reductases as well as GSSG reductase provide potential targets to support or restore redox networks.

In efforts to preserve or restore redox networks, attention must be given to diurnal variations in redox systems. The GSH and Cys redox systems, each undergoes diurnal variation, with greatest oxidation in the morning [98]. The GSH changes are delayed relative to the Cys changes and have lesser extent of variation. Additionally, the amplitude of variation of the Cys system was 1.4-fold greater in individuals >60 years compared with individuals <40 years [98]. Similarly, diurnal variations occur in oxidation of peroxiredoxins [99]. Whether such variations affect outcomes in CAD or other disease processes is unknown and warrants additional study.

## Implications for regenerative medicine

As described in the previous sections, redox theory emphasizes the importance of early life and cumulative lifelong exposures as critical determinants of health and disease and directs attention to central hubs controlling the thiol/disulfide systems, which have been linked to health behaviors, disease, and death. Redox theory also has implications for regenerative medicine, i.e. research in biologics, medical devices, and combination products to regenerate, replace, or repair tissues and organs [1,56]. Advances in stem cell research and tissue engineering have catapulted forward regenerative medicine. Yet redox theory predicts that cumulative lifetime exposures and adaptive responses will result in tissue scaffolds with molecular and macromolecular scarring, such as damaged extracellular matrix, as well as mutations, senescent cells, abnormal cell populations and epigenetic changes, which must be addressed to enable tissue regeneration [56]. Preconditioning regimens are likely to be needed to address these molecular and macroscopic impediments to efficient repair and regeneration.

The age-associated decline and failure of lung function provide an example for consideration of this important subject. A conceptual overview of barriers to regeneration in the lungs, with approximately 40 cell types and complex anatomy and cell physiology, is available [100]. A broad spectrum of lung diseases occurs with multiple molecular pathways, anatomic diversities, temporal behaviors, and relative intensities of disease phenotypes [100–104]. Consequently, effective regeneration is likely to require conditioning approaches specific for disease processes and/or personalized exposure histories. These conditioning steps are needed to clear scars and replace dysfunctional extracellular matrix, eliminate mutated and senescent cells, reset adaptive systems, and reverse epigenetic marks. This conditioning will allow tissue engineers and stem cell biologists to induce new lung regeneration niches for expansion and development of architecture and cell populations to regenerate lung function [105].

Redox theory also emphasizes the role of spatial and temporal redox control in the developmental programs. Recapitulation of polarity of O_2_ delivery, pH control and H_2_O_2_ and redox potential gradients, in organogenesis, as well as avoidance of xenobiotic chemicals affecting critical receptor signaling will be essential for full functional recovery of airway epithelium and alveolar lined spaces. Thus, an implication of redox theory is that emphasis on understanding the human exposome and exposure memory will not only enhance ability to prevent and manage human health and disease, but also help usher in the promising new approaches to regenerate, rather than stop and repair, organ system functions.

## Summary and perspective

Loscalzo et al. [106] presented a complex systems approach to disease, which anticipates many of the conclusions derived from redox theory. They note that a single genetic change in sickle cell disease results in multiple disease phenotypes and that in cardiovascular disease, multiple etiologies result in a single disease. Redox theory provides a foundation for cause–effect relationships in complex systems, starting from individual genetics and sequentially incorporating environmental effects within developmental programs, growth, and maturation. From this perspective, the exposome can be viewed as an integral of gene–environment interactions over a lifespan. This provides logic for development and function of complex systems. An individual is a complex system of cell types, tissues, and organs, which work together as a functional unit. The design of the functional unit is molded during development, growth, and maturation, through responses of subnetworks to environmental exposures. This sequence of exposures and cumulative responses determines subsequent performance of the functional unit, from cognitive to physical capabilities as well as responses to food intake, infection, and other exposures.

The interaction of organ systems within the overall network is responsible for onset of specific diseases and to the most common outcome, multimorbidity [107]. In broad terms, if the lung function is impaired, other organ systems must accommodate the decreased capabilities for O_2_ delivery, CO_2_ elimination, and respiratory pH control. Poor lung function must affect functions in the heart, peripheral vasculature, kidneys, intestines, and other organ systems. Thus, as shown by Barnett et al. [107], more than 70% of patients with COPD have other chronic health conditions. A central implication of redox theory is that multimorbidity is a general consequence of morbidity. Each organ system is dependent upon all other systems to optimize utilization of environmental resources and protect against environmental threats, so declining function of one system necessarily adds strain and decreases adaptability of other systems. The integrated nature provides stability and can obscure underlying disease etiologies. Ultimately, an atlas of network responses to common exposure–outcome relationships will help early diagnosis and development of interventions to delay or prevent decline in network functions.

More specific implications of redox theory for human health and disease are summarized in Table 2. The importance of early life exposures on health outcomes is well recognized, but details are missing. For instance, if adult size represents an adaptive influence of environment on individual genetics during development, then optimized adult health may depend more upon behaviors and exposures matched to this gene–environment interaction than to adult size, per se. Redox theory points to a need for systematic studies of effects of essential nutrients and physiologic parameters on adult phenotype as a foundation for consideration of possible effects of more recently introduced pesticides and personal care products. Without such details, personalized health management will remain tied to population averages and not progress to desired personalized level.

Redox theory also emphasizes the cumulative impact of exposures within the adaptive network structures (Table 2). A natural consequence of decline in resilience is that excesses of all types must be viewed with caution, whether those include excessive time on a sofa or excessive super-marathons. Strenuous physical conditioning, fasting, immunizations, and other common stressors warrant further study to understand when a stressor enhances resilience as opposed to causing a loss of resilience. Appropriate life-stage adjustment of behaviors and exposures can be expected to preserve resilience with age (Table 2).

The availability of CySS/GSH as a potential measure of the fitness of an individual’s redox networks has implications for interventions to improve individual resilience (Table 2). Widespread utility of CySS/GSH is limited by sample collection needs for GSH, but plasma CySS is readily measurable and provides predictive value independent of GSH. As indicated above, some options are available to limit CySS accumulation, and studies in patients with cystinuria further suggest that restriction of methionine intake could be beneficial.

Finally, the redox theory of aging reveals a need to elucidate the central principles of exposure memory, i.e. the central guidelines for lifelong exposures to optimize an individual exposome for healthy longevity (Table 2). Cellular differentiation and development in animals are ultimately linked to the benefits of having oxidizable food sources for energy and metabolism and control systems to manage O_2_ delivery, oxidative stress, and environmental threats. Simple principles of the redox code are followed in redox organization and function, and the resulting redox networks and exposure memory systems effectively account for the hallmarks of aging. A critical need exists to elucidate the hierarchy of exposure memory systems that the genome uses to sense, respond, and remember environmental resources and challenges. This includes the signaling structure to maintain mitochondrial integrity, bioenergetics, and oxidative and xenobiotic defenses, as well as optimized systems for metabolic regulation and regenerative capacity, DNA repair, and immunity.

A central focus for etiology of complex disease is refined, therefore, to a need to understand the logic for early environmental responses that affect adult health and disease risk. When viewed as an integral of gene–environment interactions over lifespan, the critical nature of these early exposures in adult disease risk is magnified. Human expo-some research is poised to elucidate these central mechanisms of exposure memory and support use of this knowledge to improve individual health and provide strategies to prevent and manage disease.

## Figures and Tables

**Table 1:** Major causes of human morbidity and mortality are associated with oxidative stress

Disease	Death (rank)	Prevalence (rank)	Condition	Prevalence (%)	Oxidative stress references
Heart disease	1	1	Hypertension	13.4	[110,111]
		5	Coronary heart disease	4.7	[112,113]
		21	Atrial fibrillation	1.4	[114]
		23	Heart failure	1.1	[115–117]
Cancer	2	14	Cancer (malignant neoplasms)	2.5	[118,119]
Mental disorders		2	Depression	8.2	[120–122]
		12	Anxiety and other neurotic, stress-related, and somatoform disorders	3.2	[123–126]
		16	Other psychoactive substance misuse	2.4	[127–129]
		27	Dementia	0.7	[130]
	6		Alzheimer’s disease	1.7	[131,132]
		28	Schizophrenia (and related non-organic psychosis) or bipolar disorder	0.7	[133,134]
Pulmonary conditions	3	4	Asthma (currently treated)	6	[135–137]
		11	Chronic obstructive pulmonary disease (COPD)	3.2	[135,137]
		36	Bronchiectasis	0.2	[138–140]
			Pneumonia		[141,142]
Stroke, cerebrovascular disease	4	18	Stroke and transient ischemic attack	2.1	[143,144]
			Trauma		[145,146]
		3	Painful condition	7.2	
Diabetes	7	7	Diabetes	4.3	[147,148]
Endocrine		8	Thyroid disorders	4.1	[149,150]
Autoimmune diseases		9	Rheumatoid arthritis, other inflammatory polyarthropathies and systematic connective tissue	3.4	[151,152]
		38	Multiple sclerosis	0.2	[153,154]
		30	Inflammatory bowel disease	0.6	[155,156]
		6	Treated dyspepsia	4.5	
Gastrointestinal diseases					
		13	Irritable bowel syndrome	3	
		17	Treated constipation	2.2	[157]
		20	Diverticular disease of intestine	1.9	
Kidney disease	9	19	Chronic kidney disease	1.9	[158,159]
Hypertension	13	22	Peripheral vascular disease	1.3	[160]
			Prostate disorders	0.9	[161,162]
Eye disease		25	Glaucoma	0.9	[163,164]
		32	Blindness and low vision	0.5	
Skin disorders		29	Psoriasis or eczema	0.7	[165,166]
		10	Hearing loss	3.4	[167–169]
Liver diseases	12	40	Chronic liver disease	0.1	[170,171]
		15	Alcohol problems	2.4	[172,173]
		33	Chronic sinusitis	0.5	
		34	Learning disability	0.3	
		35	Anorexia or bulimia	0.3	[174,175]
Infectious disease		39	Viral hepatitis	0.1	[176,177]
	8		Influenza and pneumonia		[178,179]
	11		Septicemia		
Neurological disorders	14	37	Parkinson’s disease	0.2	[180–182]
		31	Migraine	0.6	[183,184]
		26	Epilepsy	0.8	[185,186]

Morbidity data are based upon Barnett et al. [107] and mortality data are based upon Heron et al. [108,109]. References are provided for conditions with evidence for oxidative stress.

**Table 2:** Health implications of redox theory and future needs

Health implications of redox theory	

Redox theory	Implication/Limitation
Early exposures determine lifelong physical form and functional capacities	Population norms are poor guide for personalized health and disease prevention
Exposures have cumulative impact throughout life	Extremes of all types should be avoided
Differentiation and exposure memory progressively limit adaptability of redox networks	Life-stage activities and exposures can be adjusted to improve lifelong resilience
Plasma CySS/GSH provides the best available measure of healthy redox networks	Measures of plasma CySS are reliable but GSH can be overestimated due to trace hemolysis
Definition of ‘exposure memory code’ would facilitate research to erase and restore network resilience	Research lags progress in stem cell research and tissue engineering and warrants investment
**Perspective: needs for the future healthy longevity project**	
1. Optimize an individual exposome for healthy longevity/aging	
2. Elucidate the hierarchy of exposure memory systems in association with personal medical record	
3. Apply an advanced systems biology approach to integrate redox proteomics, metabolomics, (epi)genomics, and exposomics, to understand functional network responses with life (aging) and predict individual health and disease	

## Abbreviations

CAD: coronary artery disease
COPD: chronic obstructive pulmonary disease
Cd: cadmium
Cys: cysteine
CySS: cystine
*E*_h_: redox potential
GSH: glutathione
GSSG: glutathione disulfide
IL-1 beta: interleukin-1 beta
KGF: keratinocyte growth factor
keap-1: Kelch-like ECH-associated protein 1
ROS: reactive oxygen species
Trx: thioredoxin
TNF-alpha: tumor necrosis factor alpha
Nrf2: nuclear erythroid 2-related factor 2
NF-kB: nuclear factor kappa b
